# COVID-19 impact on AMR: a rapid scoping review, equity analysis and evidence gap map study

**DOI:** 10.1136/bmjgh-2024-018118

**Published:** 2025-11-29

**Authors:** Fiona Emdin, Ebiowei Samuel F Orubu, Susan Rogers Van Katwyk, Kayla Strong, Nicole Shaver, Kawsari Abdullah, Gideon Asamoah, Becky Skidmore, Emilie Chan, Mathieu J P Poirier

**Affiliations:** 1 York University, Toronto, Ontario, Canada; 2 Global Strategy Lab, York University, Toronto, Ontario, Canada; 3 Knowledge Synthesis and Application Unit, University of Ottawa, Ottawa, Ontario, Canada; 4 Independent Information Specialist, Independent Information Specialist, Ottawa, Ontario, Canada

**Keywords:** COVID-19, Health policy, Global Health, Health policies and all other topics

## Abstract

**Introduction:**

The COVID-19 pandemic is expected to have impacted many drivers of antimicrobial resistance (AMR) and compounded existing societal and health inequities. This rapid scoping review examined how three selected healthcare system factors, which we have called ‘drivers’—antimicrobial use, infection prevention and control and health system use—were affected by COVID-19 and how they have impacted resistance.

**Methods:**

Peer-reviewed searches were performed in MEDLINE, Embase and Cochrane on 19 December 2022 and updated on 25 February 2023 and 1 September 2023. Results of these searches were integrated with an initial search run on 19 October 2022, using the WHO COVID-19 Research Database. References of included studies were also searched to identify any additional relevant studies. Data on the three drivers from included studies were assessed to determine whether they influenced the emergence, spread or number of resistant infections due to antimicrobial-resistant organisms. Studies were then mapped to identify literature gaps and assessed for equity considerations and quality of evidence.

**Results:**

63 studies were analysed. Reported COVID-19 changes to antimicrobial use were associated with increased AMR burden in hospital settings. Conversely, the infection prevention and control measures implemented to reduce COVID spread may have decreased resistance in community settings. Differences in health system use during the COVID-19 pandemic may have increased resistance, although we identified knowledge gaps on COVID-19-related changes in health system use. Few studies considered equity in their analyses and no studies directly mentioned equity. All included studies had a moderate to high risk of bias.

**Conclusions:**

COVID-19 led to mixed effects on AMR, which depended on the setting and context. There is a need for more rigorous studies that examine how COVID-19 impacted the health system as well as socioeconomic determinants to provide evidence for future pandemics or health crises. Our findings also underscore the importance of integrating antimicrobial stewardship, robust infection prevention and equity-focused surveillance into pandemic preparedness to mitigate AMR risks in future public health emergencies.

**WHAT IS ALREADY KNOWN ON THIS TOPIC**

- The COVID-19 pandemic changed how antimicrobials were used; in many hospitals, antimicrobial use increased to treat secondary bacterial infections in COVID-19 patients, while at the same time, infection prevention and control measures such as lockdowns, travel restrictions and wearing face masks were implemented.
- It is unknown whether these pandemic-related changes increased or decreased antimicrobial resistance (AMR).

**WHAT THIS STUDY ADDS**

- This descriptive review found that during the COVID-19 pandemic, antimicrobial use and AMR may have increased in hospital settings and decreased in community settings.
- Very few relevant studies considered equity in their analyses.

**HOW THIS STUDY MIGHT AFFECT RESEARCH, PRACTICE OR POLICY**

- These findings imply that pandemics have the potential to both inflate and diminish AMR in hospital and community settings; however, trends are location specific and are limited by knowledge gaps and low quality of evidence.

## Introduction

 Antimicrobial resistance (AMR)—when disease-causing microorganisms such as bacteria, fungi and parasites evolve to evade antimicrobial medicines—has been identified as one of the top 10 global public health concerns by the WHO.^1^ In 2021, AMR contributed to almost 5 million deaths and was the third leading cause of death globally.^2^

The inappropriate use of antimicrobials facilitates the development and spread of AMR. The widespread misuse and overuse of antimicrobial medicines have promoted resistance in microorganisms, accelerating a selection process across human, animal, plant and environmental sectors.^3^ If left unchecked, AMR will render antimicrobial medicines ineffective, threatening our ability to treat diseases in humans, animals and plants and negatively impacting social and economic development.^4 5^

COVID-19, a novel infectious disease caused by the SARS-CoV-2 virus, emerged in late 2019 and was declared a pandemic on 11 March 2020.^6^ In the early days of the COVID-19 pandemic—with rapidly evolving treatment guidelines, media messaging and public concerns—there was an upsurge in the use of antimicrobials, some potentially inappropriate, raising concerns about the impact of this use on AMR.^7^ At the same time, infection prevention and control (IPAC) measures—both in the community with interventions such as lockdowns, travel restrictions and work-from-home requirements and in-hospital settings with improved personal protective equipment like masking—may have reduced infectious disease transmission.^8^

The COVID-19 pandemic has changed healthcare globally, and the potential positive or negative impacts of the COVID-19 pandemic on AMR have been hypothesised.^7 8^ A systematic review and meta-analysis which examined the impact of COVID-19 on AMR did not find any significant increases in AMR, although it did identify a non-significant increase in resistant Gram-negative organisms.^9^ This review found a potential link between absent IPAC measures at hospitals and increased Gram-negative AMR. To guide more robust policy and practice interventions, we need additional evidence of the impact of COVID-19 on AMR. This evidence must also include societal and health inequities that both AMR and the pandemic may have exacerbated. Both AMR and COVID-19 disproportionally affect people based on race or ethnicity,^10^ migrant status,^11^ income,^12^ gender and sexual orientation.^13^ Populations at higher risk of COVID-19 are also at higher risk of AMR infection^14^ and face additional barriers to accessing testing and treatment for AMR infections.^15^

This study aimed to identify and map evidence of the COVID-19 pandemic’s multidimensional impacts on AMR. Specifically, we examined how the COVID-19 pandemic’s impacts on antimicrobial use (AMU), IPAC practices and health system use (HSU) affected AMR emergence, AMR transmission and AMR burden. We also assessed equity considerations and gaps in the literature.

## Methods

### Study protocol

The study was designed as a rapid scoping review of the literature^16^ and was originally published as a living evidence review commissioned by the Office of the Chief Science Officer, Public Health Agency of Canada.^17^ This study was designed using the Preferred Reporting Items for Systematic Review and Meta-Analysis Protocols (PRISMA) checklist extension for Scoping Reviews.^18^

To guide reporting and analysis of the impact of COVID-19 on AMR, a framework developed by Knight *et al*^19^ that explored the intersectionality between AMR and COVID-19 was used. The Knight framework categorised the health-related aspects of COVID-19 on AMR into selected ‘drivers’ and ‘dimensions’. The framework includes three AMR drivers, AMU, IPAC and HSU, as well as three dimensions of impact—AMR emergence, AMR transmission and AMR burden. In this model, each driver can impact one or all dimensions to influence AMR. By extension, any COVID-19-related impact on a driver affects the development—emergence and transmission—or burden of AMR (table 1)*.*

**Table 1:** Knight *et al* framework of AMR drivers (AMU, IPAC and HSU) and dimensions (AMR emergence, AMR transmission and burden of AMR illness)

Drivers (COVID-19-related changes)	Dimensions		
	AMR emergence	AMR transmission	Burden of AMR illness
AMU			
IPAC			
HSU			

AMR, antimicrobial resistance; AMU, antimicrobial use; HSU, health system use; IPAC, infection prevention and control.

### Eligibility criteria

Identified studies were screened using the inclusion and exclusion criteria in table 2*.* Studies published from 2020, when the COVID-19 pandemic became widespread, to 1 September 2023 (when the last search was conducted) were included. Studies were screened to ensure they were conducted during the COVID-19 pandemic and published after 11 March 2020, when the WHO declared COVID-19 a pandemic.

**Table 2:** Inclusion and exclusion criteria of screened studies

Inclusion criteria	Exclusion criteria
The study includes one of the three drivers of AMR affected by COVID-19 as defined in the Knight framework: AMU, IPAC or HSU. The study reports the impact of at least one driver on at least one AMR dimension: emergence, transmission or burden. The emergence of new drug-resistant strains was defined as AMR emergence, the spread of antimicrobial-resistant organisms between hosts as AMR transmission and changes in the number and nature of infections due to antimicrobial-resistant organisms as AMR burden. Any quantitative study which reported a direct or indirect measure of association between at least one driver and one AMR dimension before and during the COVID-19 pandemic.	The study did not report on at least one of the three selected drivers. The study did not report on the impact of at least one driver on at least one AMR dimension. The study did not report any measure of association between a driver and dimension before and during the COVID-19 pandemic. The study is a non-systematic review, case report, case series, survey, modelling study, commentary, letter, conference abstract or qualitative study. The study is not published in English between March 2020 and September 2023.

AMR, antimicrobial resistance; AMU, antimicrobial use; HSU, health system use; IPAC, infection prevention and control.

Study identification, title and abstract screening, study selection and data extraction were completed by a single reviewer (FE) (online supplemental appendix 1: table S1). A team of three reviewers (NS, EC, KA) then validated 30% of single-reviewer screenings at both the abstract and full-text stages and did not identify any missed studies.

### Search strategy and information sources

An initial search was run on 19 October 2022, in the WHO COVID-19 Research Database,^20^ which pulls records from several databases. The results of this search were integrated with the findings from the detailed search strategy, which was developed by an experienced information specialist (BS) in consultation with the review team (online supplemental appendix 2). The MEDLINE strategy was peer reviewed before execution by another senior information specialist using the Peer Review of Electronic Search Strategies (PRESS) checklist.^21^ The search strategy was developed to capture any study which examined the impact of COVID-19 on AMR in any setting, such as within a community, hospital or globally. Search terms related to AMR, antimicrobials and the COVID-19 pandemic were created using a combination of Medical Subject Headings terms (eg, ‘COVID-19’, ‘Drug Resistance, Microbial’, ‘Antimicrobial Stewardship’) and keywords (eg, ‘2019-nCoV’, ‘antibiotic resistance’, ‘multidrug resistance’). Using the multifile option and deduplication tool available on the Ovid platform, the following databases were searched: Ovid MEDLINE ALL, Embase, EBM Reviews–Cochrane Database of Systematic Reviews, and EBM Reviews—Cochrane Central Register of Controlled Trials. All searches were performed on 19 December 2022 and updated on 25 February 2023 and 1 September 2023. Results were limited to studies published from 2020 up to the search date to ensure relevance to the current pandemic. Records were downloaded and deduplicated using EndNote V.9.3.3 (Clarivate Analytics) and uploaded to Covidence.

The references of identified studies were also searched using a snowballing approach to identify additional eligible studies.

### Data extraction and data items

Data were extracted by a single reviewer (FE). Data items extracted were the author, year, type of study, dates of data collection, setting (hospital or community), study location (country of data collection reported in study), microbe/pathogen type(s), driver (and details about the driver, if applicable) and dimension.

### Synthesis of results

Results were summarised descriptively. The study search and selection process were summarised using a PRISMA flow chart.^22^ Studies were grouped for synthesis using the Knight *et al*^19^ framework. This same framework was also used to generate an evidence gap map showing which drivers and dimensions are not well examined by literature.

Study data were analysed and reported using AMR dimensions stratified by driver and setting. Dimensions were AMR burden (a change in the number of infections due to antimicrobial-resistant organisms), AMR emergence (emergence of drug resistance strains including change in resistance genes) and AMR transmission (any measure of horizontal transmission). Drivers were changes in AMU (eg, defined daily doses, tonnes of antimicrobials used), IPAC (eg, hand sanitiser use, masking) and HSU (eg, a change in number of diagnostic tests or referrals). In the descriptive analysis, studies were grouped by the year in which data were collected (2020, 2021 or 2022) to examine when the impacts of AMR became apparent during the COVID-19 pandemic.

### Risk of bias

Risk of bias assessments for non-randomised studies (such as interrupted time series (ITS) designs) were completed with the Risk of Bias in Non-Randomized Studies of Interventions tool (ROBINS-I).^23^ The Newcastle-Ottawa Scale (NOS)^24^ was used to evaluate cohort studies, and finally, environmental sampling studies were evaluated using the Collaboration for Environmental Evidence Critical Appraisal Tool.^25^ Four authors (NS, KA, EC, GA) conducted the risk of bias assessment (single assessor, with verification) of included studies, and studies were classified as low, moderate or high risk of bias (or serious risk when using ROBINS-I tool).

### Equity

The inclusion of health inequities in each study was analysed using the PROGRESS-Plus framework (online supplemental appendix 1: table S2). The PROGRESS-Plus framework identifies characteristics that stratify health opportunities and outcomes^26^ like gender and sex, race, ethnicity, culture, language, religion, education, socioeconomic status, occupation and place of residence. ‘Plus’ factors include those used to refer to personal characteristics associated with discrimination like disability, features of relationships such as parents who smoke and time-dependent relationships such as time spent in hospital.

## Results

**Figure 1:**
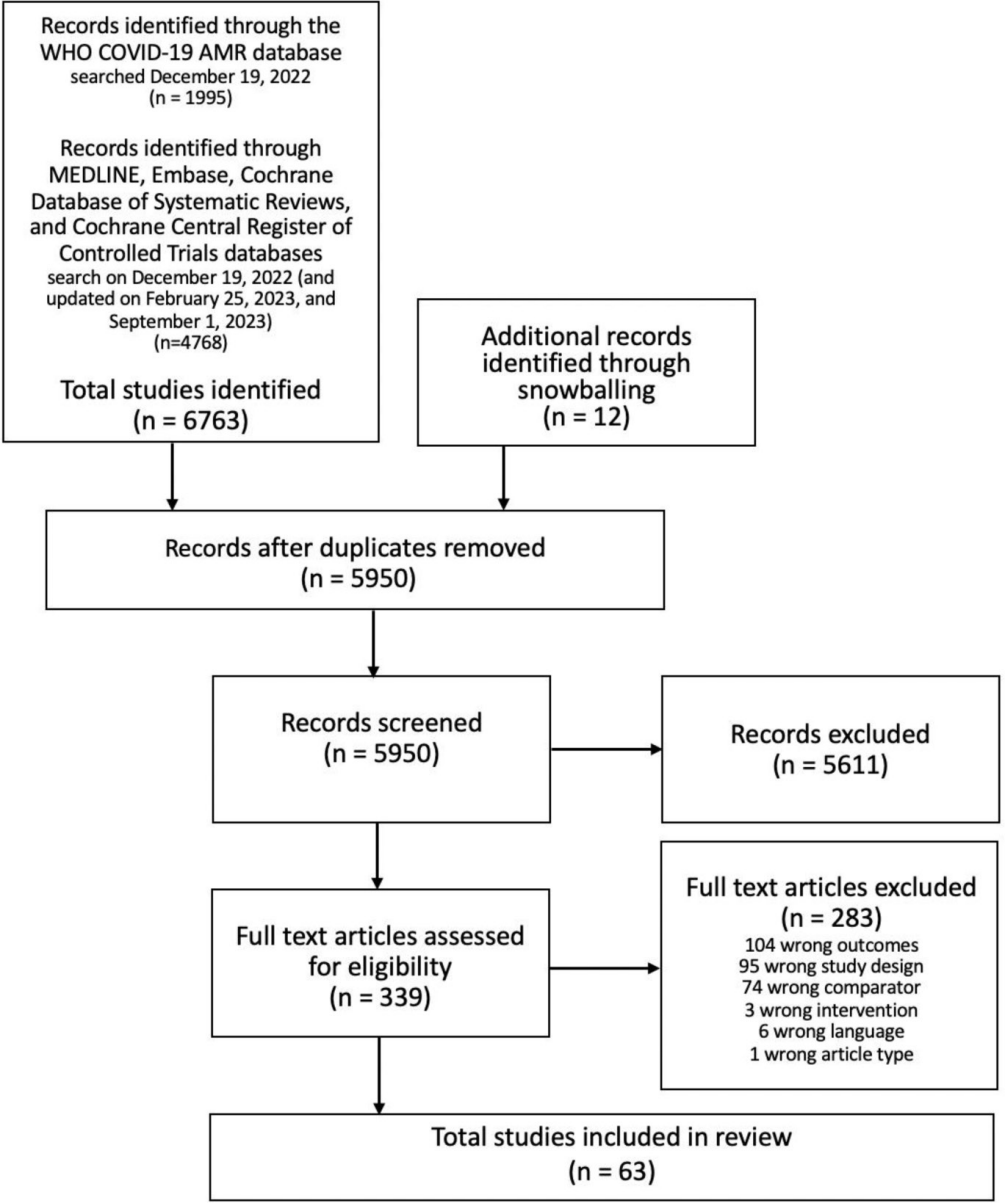
Preferred Reporting Items for Systematic Reviews and Meta-Analyses (PRISMA) flow chart depicting study selection process for this review. AMR, antimicrobial resistance.

The search of the four databases and the original search, which was run in the WHO COVID-19 Research Database,^20^ produced 63 studies. The last search completed was 1 September 2023. After removing duplicates, 5950 studies were screened at title and abstract level, and 339 were screened at full text. Of these, 51 studies met the criteria for data extraction. An additional 12 studies were identified by hand searching references of the 51 studies for other relevant studies. Thus, a total of 63 studies were included for data extraction^27^,^89^ (figure 1).

### Characteristics of studies

In descending frequency, study designs were ecological (79%, 50/63), ITS (16%, 10/63), retrospective cohort (3%, 2/63) and retrospective case–control (2%, 1/63). Most studies were hospital based or used hospital admission data. 42 studies (67%) were conducted in hospitals. 11 studies (17%) were in community settings,^43 54 55 57 60 61 71 74 75 77 80^ two studies (3%) were from environmental settings,^82 89^ and eight studies (13%) collected data from both hospital and community settings.^28 38 46 50 53 59 62 72^ The effect of IPAC measures in response to COVID-19 on the burden of AMR was, respectively, the most studied driver and dimension. Concerning the three drivers, slightly more than half of the studies (56% (35/63)) collected data on IPAC,^37^,^5557^ 41% (26/63) on AMU,^27^,^3638 51 56 61^ and 9% (6/63) on HSU^73^,^7681 88^. The most studied dimension of impact was AMR burden, with 58 studies (92%) reporting on this dimension^27^,^81^: two studies considered AMR emergence (3%, 2/63)^82 89^ and three on AMR transmission (5%, 3/63).^83^,^85^ In slightly more than half of the studies (53% (34/63)), the observation period ended in 2020.^3031 33^,^35 38^ A full list of study characteristics is available in table 3.

**Table 3:** Characteristics of studies included in the analysis of the impact of COVID-19 on AMR

Author	Country or region	AMR dimension	AMR driver	Setting	Change to driver	Change to AMR
Tham *et al*^78^	Australia	Burden	IPAC	Hospital	↑	No change
Mannathoko *et al*^53^	Botswana	Burden	IPAC	Community, Hospital	­↑	↓
de Carvalho Hessel Dias *et al*^33^	Brazil	Burden	AMU	Hospital	­↑	­↑
Freire *et al*^64^	Brazil	Burden	AMU	Hospital	­↑	No change
Pereira *et al*^68^*	Brazil	Burden	AMU	Hospital	↑ Azithromycin ↓ All other antibiotics	­↑
Allel *et al*^27^*	Chile	Burden	AMU	Hospital	­↑	­↑
Chen *et al*^38^	China	Burden	IPAC, AMU	Community, Hospital	­↑ IPAC ↓ AMU	­↑ MRSA No change in all other pathogens (VRE, CRE, CRA, or CRPA)
Yang *et al*^79^	China	Burden	IPAC	Hospital	­↑	­↑
Zhu *et al*^88^	China	Burden	IPAC	Community	­↑	↑ MRSA and CRE ↓ E. coli and Klebsiella pneumoniae to third-generation cephalosporins
Hurtado *et al*^65^*	Columbia	Burden	AMU	Hospital	­↑	Varied (increased, decreased and no-change) by bug-drug combination
Gisselø *et al*^84^	Denmark	Transmission	IPAC	Hospital	­↑	↓
Lemenand *et al*^43^	France	Burden	IPAC	Community	­↑	↓
Dapper *et al*^46^*	Germany	Burden	IPAC	Community, Hospital	­↑	No change
Ullrich *et al*^59^	Germany	Burden	IPAC	Community, Hospital	­↑	↓
Meyer Sauteur *et al*^54^*	Global	Burden	IPAC	Community	­↑	↓
Petrakis *et al*^76^*	Greece	Burden	HSU	Hospital	Infectious disease consultations decreased, and telephone consultations increased	­↑
Cheng *et al*^28^*	Hong Kong	Burden	AMU	Community, Hospital	↓	No change
Wong *et al*^69^*	Hong Kong	Burden	IPAC	Hospital	­↑	↑ CRA No change in MRSA and ESBL-producing Enterobacterales infections
Dutta *et al*^47^	India	Burden	IPAC	Hospital	­↑	↓
Jani *et al*^82^	India	Emergence	IPAC	Community	­↑	↓
Kumar *et al*^89^	India	Emergence	AMU	Community	­↑	­↑
Zaveri *et al*^45^	India	Burden	IPAC	Hospital	­↑	↓
Aldeyab *et al*^62^	Northern Ireland	Burden	AMU	Community, Hospital	No change (hospital), ↓ Community	No change
Bentivegna *et al*^39^	Italy	Burden	IPAC	Hospital	­↑	↓
Bussolati *et al*^34^	Italy	Burden	AMU	Hospital	↓	No change
Gaspari *et al*^49^*	Italy	Burden	IPAC	Hospital	­↑	↓
Meschiari *et al*^29^*	Italy	Burden	AMU	Hospital	↓ trend (No significant difference in antimicrobial consumption in any antibiotic class)	Non-significant ↑
Micozzi *et al*^83^	Italy	Transmission	IPAC	Hospital	↑	↓
Pascale *et al*^85^	Italy	Transmission	IPAC	Hospital	­↑	No change in CPE ↑ CRA
Russotto *et al*^70^*	Italy	Burden	IPAC	Hospital	­↑	↓ MRSA ↓ CRE (associated with IPAC measures)
Shbaklo *et al*^56^*	Italy	Burden	AMU	Hospital	↑ Fourth- and fifth-generation cephalosporins and piperacillin–tazobactam ↓ Fluoroquinolone use	­↑
Tedeschi *et al*^61^	Italy	Burden	AMU	Community	↓	­↓
Zuglian *et al*^73^	Italy	Burden	HSU	Hospital	Increase in beds and fewer numbers of nurses per patients in ICUs	­↑
Endo *et al*^37^*	Japan	Burden	IPAC, AMU	Hospital	↑ IPAC ↑ AMU	No change in MRSA and quinolone-resistant *Escherichia coli*, ↑ penicillin-resistant *Streptococcus pneumoniae*
Hibiya *et al*^50^	Japan	Burden	IPAC	Community, Hospital	­↑	No change
Imoto *et al*^51^*	Japan	Burden	IPAC, AMU	Hospital	↑ IPAC ↑ AMU	Non-significant ↓
Sasaki *et al*^36^*	Japan	Burden	AMU	Hospital	­↑	↑ ESBL-producing Enterobacterales, No change in MRSA
Chamieh *et al*^31^	Lebanon	Burden	AMU	Hospital	↓	↓
López-Jácome *et al*^87^	Mexico	Burden	AMU	Hospital	­↑	­↑
Ochoa-Hein *et al*^42^	Mexico	Burden	IPAC	Hospital	­↑	No change in MRSA, CPE, ESBL, Ampicillinase C producing bacteria and CRE, ↓ multidrug resistant P. aeruginosa
Soto Hernández *et al*^81^*	Mexico	Burden	HSU	Hospital	Reduced number of elective surgeries	No change
Mughini-Gras *et al*^55^*	Netherlands	Burden	IPAC	Community	­↑	↓
Zondag *et al*^80^	Netherlands	Burden	IPAC	Community	­↑	Increase in low-level azithromycin resistance but also ceftriaxone susceptibility in *Neisseria gonorrhoeae*
Alao *et al*^74^*	Nigeria	Burden	HSU	Community	Reduced access to services, testing and treatment	­↓ from 2016 to 2021, ↑ in 2022
Mączyńska *et al*^66^*	Poland	Burden	AMU	Hospital	­↑	­↑
Teixeira *et al*^58^	Portugal	Burden	IPAC	Hospital	↓	↓
Vyazovaya *et al*^75^*	Russia	Burden	HSU	Community	Undertesting, reduced resources	No change
Wee *et al*^41^	Singapore	Burden	IPAC	Hospital	­↑	No change in CRE ↓ MRSA
Kastrin *et al*^77^*	Slovenia	Burden	AMU	Community	↓	↓
Jeon *et al*^86^*	South Korea	Burden	AMU	Hospital	­↑	­↑
Chang *et al*^63^*	Taiwan	Burden	IPAC, AMU	Hospital	↑ IPAC ↑ AMU	No change
Fukushige *et al*^48^	Taiwan	Burden	IPAC	Hospital	↑	No change
Lin *et al*^60^*	Taiwan	Burden	IPAC	Community	­↑	No change
Lo *et al*^40^	Taiwan	Burden	IPAC	Hospital	­↑	↓
Tang *et al*^57^*	Taiwan	Burden	IPAC	Community	­↑	↓
Guven *et al*^44^	Turkey	Burden	IPAC	Hospital	­↑	No change
İpek *et al*^52^	Turkey	Burden	IPAC	Hospital	­↑	↓
Önal *et al*^67^*	Turkey	Burden	AMU	Hospital	­↑	­↑
Zhu *et al*^72^*	United Kingdom	Burden	HSU	Hospital	Increased numbers of critically ill patients, prolonged ICU admission	↑ Hospital
Bauer *et al*^32^*	United States	Burden	AMU	Community, Hospital	­↑ (Hospital)	↓ Community ↑ Hospital
Bork *et al*^30^	United States	Burden	AMU	Hospital	­↑	No change
Hosseini *et al*^71^*	United States	Burden	IPAC	Community	­↑	No change in duodenal microbial alpha diversity, but beta diversity (associated with resistance) was significantly different
Santos *et al*^35^	United States	Burden	AMU	Hospital	No change in vancomycin, linezolid, ceftolozane–tazobactam and colistin Decreased meropenem and daptomycin Increased ceftazidime–avibactam	No change in CRA, MRSA and MDR Pseudomonas aeruginosa ↑ ESBL-producing Enterobacterales, VRE and CPE

Additional study details can be found in online supplemental appendix 1: table S1.

↑ denotes increasing, ↓ decreasing.

* Indicates studies where observational period continued beyond 2020.

AmpC, ampicillinase C; AMR, antimicrobial resistance; AMU, antimicrobial use; CPE, carbapenemase-producing Enterobacteriaceae; CRA, carbapenem-resistant *Acinetobacter baumannii*; CRE, carbapenem-resistant Enterobacteriaceae; CRPA, carbapenem-resistant *Pseudomonas aeruginosa *; ESBL, extended-spectrum beta-lactamase; HSU, health system use; ICU, intensive care unit; IPAC, infection prevention and control; MDR, multidrug resistant; MRSA, methicillin-resistant *Staphylococcus aureus*; VRE, vancomycin-resistant *Enterococcus*.

### COVID-19 impact on AMR box

#### AMR emergence

The results of COVID-19 on AMR emergence were mixed. Only two studies considered this dimension, both of which examined changes in the prevalence of antimicrobial-resistant genes before and after COVID-19 in waterways in India. Both studies used data from 2020. One study reported increased rates of resistance, which was attributed to higher rates of AMU^89^; the other study reported reduced resistance, which was attributed to stringent IPAC measures and lower numbers of mass bathing events due to lockdowns.^82^

#### AMR transmission

Of the three studies on AMR transmission, two^83 84^ reported reduced transmission of resistant pathogens due to increased IPAC measures, while one reported no change in carbapenemase-producing Enterobacteriaceae.^85^ No significant difference in carbapenemase-producing Enterobacteriaceae colonisation or infection rates during 2020, but observed an increase in carbapenem-resistant *A. baumannii*, was reported by one Italian multicentre study (2019–2020).^85^ These measures included masking, gloves, gowns, increased visitor restrictions and reduced transfers between wards.^85^ Conversely, an ITS, multicentre analysis from Italy using data from 2020 reported reduced colonisation and infection with vancomycin-resistant *E. faecium* before and during the pandemic.^84^ Similarly, in a Danish centre, vancomycin-resistant *Enterococcus faecium* outbreaks were found to have decreased by 10-fold in 2020.^84^

#### AMR burden

Most hospital studies that focused on AMU or HSU reported increased AMR burden, while studies that focused on IPAC interventions reported decreased AMR burden. The impact of each driver on AMR burden is described below.

In studies that examined AMU or HSU as a primary focus, most reported increased AMR burden, although they did not always directly attribute changes in AMU and HSU as causal. Of the 20 hospital-based studies, 16 (80%) reported increases in AMU,^2730 33 35^,^37 51 56 63^ two (10%) reported reduced AMU^31 34^ and two (10%) saw no change in AMU.^29 37^ Among the 16 studies with increased AMU, nine (56%) reported increased AMR—six studies (37%) in all pathogens^27 56 67 68 86 87^ and three studies (19%) in a single pathogen only.^33 37 66^ Comparatively, three studies (19%, 3/16) reported no change in AMR^30 63 64^, one (6%, 1/16) reported decreased AMR^51^ and three (19%, 3/16) reported mixed resistance—both increased and decreased AMR—among the pathogens they examined.^35 36 65^ Increased AMU was frequently reported alongside increased AMR from settings with increased healthcare strain during the pandemic, such as COVID-19 referral or intensive care unit (ICU) hospitals, where both AMU and AMR were monitored.^33 34 37 39 67^ In the community, all four studies reported decreased AMU with varying rates of AMR.^28 61 62 77^ Two studies (50%), an Italian study using 2020 data and investigating change in resistance among strains of Enterobacterales and a Slovenian study using data until 2022 and investigating change in *Streptococcus pneumoniae* to penicillin, reported reduced resistance.^61 77^ One study (25%) from Northern Ireland reported no change in methicillin-resistant *Staphylococcus aureus* (MRSA).^62^ Finally, another study (25%) from Hong Kong reported increasing rates of MRSA but no other bloodstream infections.^28^

Studies that focused on IPAC interventions generally associated these measures with reduced AMR burden. Overall, 61% (19/31) of studies reported reduced AMR burden from improved IPAC practices in both hospitals and the community.^3139^,^43 45 47 49 51^ Of the hospital-based studies, 67% (12/18) reported that reduced AMR corresponded to improved IPAC measures during COVID-19^39^,^4245 47 49 51^. The authors attributed this to pandemic-related IPAC measures, including masks, face shields, disposable gowns and hand hygiene. Likewise, in the community-focused studies, 54% (7/13) examined community IPAC measures and reported a reduction in AMR^4353^,^55 57 59 88^. These IPAC measures differed from hospital settings and included physical distancing, stay-at-home orders, school and shop closures, cancellation of mass events, closing borders and travel restrictions.

In studies examining HSU, increased strain on health systems (reduced access to consultations, increased ICU admissions) was generally associated with increased AMR. Two hospital studies (67%, 2/3) reported increased AMR from reduced infectious disease consultations and increased ICU admissions.^73 76^ A single study considered hospital changes in MRSA and reported that hospital MRSA incidence increased during the pandemic.^72^ Comparatively, one study reported no change in AMR^81^ despite the reduced number of surgical procedures at the hospital. Within community settings, a study which used data from 2022 (later in the pandemic) reported an increase in multidrug-resistant tuberculosis (MDR-TB) which was attributed to reduced access to treatment, testing and detection.^74^

Overall, studies ending later in the pandemic (2021 and 2022) were more likely to report a change in AMR burden—typically increases—compared with studies using 2020 data, which more often found no change. Specifically, 81% and 88% of studies concluding in 2021 and 2022, respectively, reported AMR changes, compared with studies ending in 2020.^30 34 44 48 50 62 64 78 85^

### Evidence gaps

**Figure 2:**
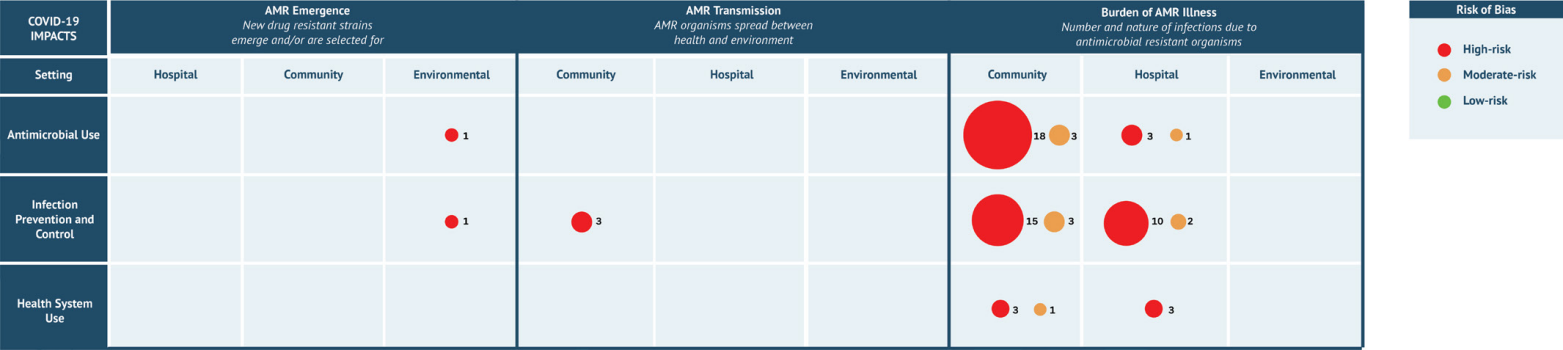
Map of study classification in accordance with the Knight *et al* framework and risk of bias. Bubble size reflects the number of studies, while bubble colour reflects the risk of bias assessment. Numbers reflect the number of studies in each bubble. AMR, antimicrobial resistance.

To identify evidence gaps, drivers and dimensions in the 63 identified studies were mapped according to Knight *et al* framework^19^ and by setting (figure 2). No included studies investigated how AMU changes may drive AMR transmission or how changes in how health systems were used may have driven AMR transmission or emergence.

### Risk of bias

Using the risk of bias assessment tools (ROBINS-I tool for non-randomised studies, NOS for cohort studies and the Collaboration for Environmental Evidence Critical Appraisal Tool for environmental studies), an overall rating of ‘moderate’ was found for 10 studies^28 32 34 38 47 53 58 64 76 78^ and a ‘high’ or ‘serious’^2729^,^31 33 35^ risk of bias was found for the remainder^53^ of the studies (online supplemental appendix 1: figures S1–S3).

Most ITS studies did not adjust for the months and time of year AMR was assessed; seasonality has been linked to infection risk for enteric pathogens like salmonella and the activity of many respiratory pathogens. They also did not provide a rationale on what date was selected as the interruption point (ie, when the pandemic started depends on the region) or on what date was chosen for post-pandemic AMR monitoring. Additionally, several studies failed to account for different follow-up times between participants. Although the laboratory methods were generally well described, sampling methodologies and surveillance systems used to obtain data on antimicrobial-resistant strains were poorly reported in many studies. Finally, the effect of bias was difficult to judge for missing outcome data, as the proportion of missing data or participants excluded was poorly reported across studies.

Within cohort studies, there was potential for bias when considering the representativeness of the exposed cohort, determining whether the outcome of interest was present at the start of the study, and choosing an appropriate length for study follow-up. Environmental sampling studies did not control for environmental confounders that may have changed unrelated to the pandemic. Although most studies reported sampling locations and laboratory procedures were the same in pre-pandemic and post-pandemic periods, there was insufficient reporting of how the environmental samples were obtained.

### Equity

Regarding the PROGRESS-Plus framework, most included studies did not report equity factors. 29 (46%) studies^2729 32 34 38 42 43 47^,^49 52^ collected data on at least one PROGRESS-Plus characteristic (online supplemental appendix 1: table S2); however, no study directly mentioned social determinants of health or equity. Most of the studies which did collect data on at least one characteristic collected only basic information, such as sex/gender (26/29) or age (28/29), without collecting other factors which contribute to social determinants of health use such as place of residence, race, ethnicity, culture or language.

## Discussion

This review, originally commissioned as a living evidence synthesis, mapped the impact of COVID-19 on AMR, the mechanisms by which it exerts impact, and knowledge gaps using the Knight *et al* framework.^19^ All included studies were assessed for quality of evidence and equity considerations.

### COVID-19 impact on AMR emergence and transmission

The paucity of studies examining the impact of COVID-19-related changes in AMU, IPAC and HSU on AMR emergence and transmission limits our ability to discuss these dimensions. However, it became clear that examining drivers of AMR in isolation provides an incomplete picture of emergence and transmission. We identified two studies which examined the dimension of emergence; metagenomics showed a reduction in infection and drug resistance genes in Indian rivers, which they attributed to restrictive IPAC measures like lockdowns,^82^ while a study from a different Indian river system reported an increase in AMR genes which they attributed to increased environmental antimicrobial pollution and AMU because of COVID-19.^89^ These findings underscore the need to examine drivers collectively.

### COVID-19 impact on AMR burden

The pandemic appears to have produced both positive and negative effects across AMR dimensions, and the overall impact on AMR depends on both the drivers and the setting being examined. A 2023 systematic review and meta-analysis also did not find significant changes in antibiotic resistance during the COVID-19 pandemic.^9^

In hospital settings, especially in ICU or COVID-19 referral settings, increased antibiotic use in COVID-19 patients may have increased AMU. Although other systematic reviews and meta-analyses have reported bacterial and fungal coinfections and secondary infections to be present at low rates in hospitalised COVID-19 patients,^90^ many still received antibiotics. One review reported that 59% of hospitalised COVID-19 patients in high-income countries and 89% in low- and middle-income countries received antibiotics.^91^

In community settings, lower reported AMU rates might result from other drivers. For example, IPAC measures such as physical distancing and masking may have contributed to a reduced burden of resistant respiratory diseases in the community.^57^ In Spain, the implementation of personal protection measures against COVID-19 transmission coincided with the lowest rates of antibiotic prescriptions.^92^ Similarly, HSU impacts in hospital settings, such as raised thresholds for seeing general practitioners for symptoms, led to reduced elective procedures and a shift to telemedicine,^93^ which may have lessened AMU use.

A study following patients with *c*hronic lymphocytic leukaemia reported a decreased incidence of bacterial and mixed infections among patients during the pandemic, likely due to pandemic IPAC measures.^94^ In both settings, IPAC measures may have reduced AMR transmission.^8^ This is especially notable for respiratory pathogens, including MDR-TB, which is one of the greatest contributors to the global AMR burden.^4^

Changes to HSU, including limited capacity to provide services and diagnosis for community-acquired diseases and reduced vaccination coverage globally,^95^ may have increased AMR. For example, global uptake of the third dose of the diphtheria, tetanus toxoid and pertussis vaccine and first measles vaccine dose fell significantly during 2020^96^. This decline occurred alongside reductions in diagnostic and preventive care access, which may have increased the likelihood of secondary bacterial infections and vaccine-preventable bacterial infections (eg, *S. pneumoniae*, *Haemophilus influenzae*), which often require antibiotic treatment and could accelerate AMR emergence.^97^

We found that studies with periods of observation ending in 2020 were more likely to find no change in resistance than those ending in 2021 or 2022. Given that the relationship between drivers and AMR dimensions is not direct, studies ending in 2020 may have been less likely to capture changes in resistance. US surveillance data from 2021 reported a rebound in antimicrobial prescribing and rising resistance rates.^98^ England also reported a decline and then an increase in AMR bacteraemia burden from 2019 to 2023.^99^ As more studies are released with periods of observation ending later, pandemic-related AMU and AMR trends may continue to emerge.

### Equity

Individual, social and structural determinants of health influence the transmission and burden of AMR. Although most studies in our review did not assess these factors, other studies have demonstrated the importance of addressing these factors. For example, many of the populations that are at higher risk of AMR^14^ are also disproportionately affected by COVID-19.^10^ For example, black, Asian and minority ethnic populations in the UK have been shown to face worse health outcomes when infected with COVID-19.^100^ In Canada, COVID-19 and related measures compromised access to sexually transmitted and bloodborne infection (STBBI) prevention, testing and treatment services, as well as harm reduction and substance use treatment services for key populations at higher risk of resistant STBBIs, such as men who have sex with men and people who use drugs.^15^ These populations may have been at higher risk of contracting AMR infections, less likely to get diagnosed and less likely to receive timely treatment for these infections during the pandemic. Some countries also faced limited or reduced access to vaccinations, reduced access to laboratory materials and reduced staff availability—all of which may drive inequitable AMR transmission.^101^ Future studies identifying the equitable factors that might influence these AMR risks are needed.

### Implications for future pandemics

Our findings and recommendations are relevant to the COVID-19 pandemic and to future pandemics and other public health emergencies. The observed effects of IPAC measures in reducing AMR in community settings and the potential for increased AMR from AMU and healthcare service disruptions in hospitals are critical areas for intervention during future pandemics. Strengthening surveillance systems to monitor AMR trends in real time, ensuring rational antibiotic use even during health system strain and maintaining core IPAC strategies should be prioritised in pandemic preparedness plans. Additionally, future AMR research and monitoring should systematically incorporate socioeconomic and structural determinants of health to identify and address inequitable impacts during public health emergencies. These steps will help build resilient health systems capable of mitigating AMR risks during future pandemics.

### Evidence gaps

Unsurprisingly, most of the included studies in the review were focused on AMR burden and individual drivers. We identified no studies which examined change in AMU as a driver of AMR transmission or change in HSU as a driver of AMR transmission or emergence; additional research targeting these knowledge gaps is especially needed. Studies considering multiple drivers are also needed. Modelling and/or genomic studies are needed to provide evidence for AMR emergence and transmission in the context of COVID-19figure 2 .

### Limitations

This was an initial rapid scoping review, followed by two 6-month updates, with screening conducted by a single reviewer, increasing the risk of missing relevant studies, but this risk was partially mitigated by a second reviewer validating 30% of screening decisions. Many included studies were assessed as having a ‘serious’ risk of bias, limiting the certainty of synthesised findings. Some included study designs were not set up for empirical evaluation of change in AMR dimension, which may also affect the certainty of data. Additional high-quality research with appropriate adjustment for confounding factors and clear, repeatable reporting is required to increase confidence in the conclusions drawn from these studies and make future statements related to causation. Additionally, pandemic-related reductions in laboratory capacity and clinical testing likely resulted in underestimation of AMR trends in some settings. Also, few studies examined AMR emergence and transmission dimensions in depth, limiting our understanding of the complex interplay between drivers. Finally, while this review captures changes to AMU, IPAC and health system utilisation during the COVID-19 pandemic (2020–2023), we acknowledge that the full impact of these changes on AMR may take longer to emerge and may not yet be fully observable within the assessment period.

To improve future pandemic response and surveillance capacity, there is a need for high-quality, longitudinal research that integrates AMR burden, emergence and transmission dimensions, with clear adjustment for confounding factors and use of both phenotypic and genotypic resistance data. Standardised methods for reporting AMR trends that account for these potential biases are needed.

## Conclusion

COVID-19 and the associated public health measures implemented in its wake led to mixed effects on AMR. There may have been increased antibiotic use and increased AMR in hospital settings during COVID-19. Conversely, the IPAC measures implemented to check COVID-19 may have decreased AMR in community settings. There is a need for robust evidence to understand the net impact of AMR and strengthen health systems for future public health emergencies. AMR research, which collects data on socioeconomic, sociodemographic, social and structural determinants of health, is needed to allow researchers and policymakers to identify and address the potential inequitable AMR impacts, especially during public health emergencies like COVID-19 and during future pandemics.

## Supplementary material

10.1136/bmjgh-2024-018118 online supplemental file 1

## Data Availability

All data relevant to the study are included in the article or uploaded as supplementary information.
