# Supplementary material for: COVID-19 impact on AMR: a rapid scoping review, equity analysis and evidence gap map study
Source: BMJ Glob Health. 2025 Nov 29;10(11):e018118. doi: 10.1136/bmjgh-2024-018118 (PMC12666195; doi:10.1136/bmjgh-2024-018118)
Supplement: online supplemental file 1 [file bmjgh-10-11-s001.docx]

## **APPENDIX 1**

*Table S1. Characteristics of studies included in analysis of the impact of COVID-19 on AMR.*

| Author, Year | Country or region | Type of study | Brief description of the study | Dates of data collection | Setting | Pathogen type(s) reported, measure of AMR reported and change to AMR |
| --- | --- | --- | --- | --- | --- | --- |
| Alao 2022 | Nigeria | Ecological | Determined the trends in rifampicin-resistant tuberculosis between the pre-COVID and COVID era. | 2016 - 2022 | Community | The incidence of rifampicin-resistant tuberculosis declined substantially from 2016 to 2021 (pre-COVID-19) but rose exponentially in 2022 (COVID-19 era). |
| Aldeyab 2023 | Northern Ireland | Interrupted Time Series | Examined change in antibiotic use and Gram-positive and Gram-negative pathogens from primary and secondary healthcare settings in Northern Ireland before and during the pandemic. | 2015-2020 | Community and Hospital | In the hospital setting, the mean total hospital antibiotic consumption did not change during the pandemic. The number of MRSA cases remained the same. In primary care, the mean total antibiotic consumption during the COVID-19 pandemic was lower than before the COVID-19 pandemic. The incidence of Gram-positives (including MRSA) did not change. |
| Allel 2023 | Chile | Interrupted Time Series | Evaluated intravenous antibiotic use and frequency of CRE pre and post COVID-19 at a tertiary hospital. | 2018-2022 | Hospital | Compared with pre-pandemic, antibiotic use significantly increased after the pandemic onset for broad-spectrum β-lactams, carbapenems, and colistin. The frequency of CP-CRE increased during the pandemic. |
| Bauer 2022 | United States | Retrospective cohort analysis | Evaluated rates of AMU and AMR events before and during the SARS-CoV-2 pandemic from 271 US facilities. | 2019 - 2021 | Hospital | The percentage of admissions prescribed antibacterial therapy was significantly higher during the pandemic than during the pre-pandemic period. During the pandemic, AMR rates among community-onset infections were lower than pre-pandemic levels, whereas AMR rates for hospital-onset infections were higher. |
| Bentivegna 2021 | Italy | Retrospective case-control | Examined the incidence of MDR infections using pandemic-related preventive measures in a single hospital. | 2017 - 2020 | Hospital | IPAC measures were higher during the pandemic. Compared to pre-pandemic years, a significant reduction in the incidence of total MDRO infections was observed during the pandemic. |
| Bork 2020 | United States | Interrupted time series | Examined MDR gram-negative acquisition and AMU before and during COVID-19 at an academic hospital. | 2019 - 2020 | Hospital | MDR gram-negative incidence did not differ significantly during the 2020 post-onset period compared to the same period in 2019. Total antibiotic use was higher after the onset of COVID-19 compared to a similar calendar period in 2019. |
| Bussolati 2022 | Italy | Retrospective cohort | Compared infections and antibiotic use in a cohort of patients admitted to the ICU to one from the year before the pandemic during the same period at a hospital. | February 2019 - April 2020 | Hospital | A comparable incidence of HAIs and MDR isolations was found in the two groups. ICU prescription of antimicrobial therapy changed and significantly decreased during the pandemic. |
| Chamieh 2021 | Lebanon | Ecological | Analyzed the antimicrobial susceptibilities of blood isolates and antimicrobial consumption before and during the COVID-19 pandemic. | January 2015 - December 2020 | Hospital | The isolation density of CRE BSI and vancomycin-resistant *E. faecium* BSI decreased. Antimicrobial consumption also decreased during COVID-19. |
| Chang 2023 | Taiwan | Ecological | Measured usage of antimicrobial agents and HAI density of five major MDR bacteria at a medical centre before and during COVID-19. | 2017-2021 | Hospital | Prevention and control measures were implemented to combat COVID-19. Antibiotic consumption was significantly increased during the pandemic period. There was no significant change in HAI density in MRSA, VRE, CRA, CRKP, and CRPA, comparing the pandemic to the pre-pandemic period. |
| Chen 2021 | China | Ecological | Examined the effect of the COVID-19 prevention and control requirements on HAI and CAI in China during 2018, 2019, and 2020. | 2018 - 2020 | Community and hospital | Analysis of HAI by MDROs indicated that MRSA infections were more common in 2020 than in 2018 and 2019, but there were no significant changes in infections by VRE, CRE, CRA, or CRPA. |
| Cheng 2022 | Hong Kong | Ecological | Examined blood cultures of patients admitted to public hospitals collected by the Hospital Authority in Hong Kong for the last 10 years. | 2012 - 2021 | Community and hospital | Mean episodes of community-onset bacteremia due to both MSSA and MRSA per year were higher during two pandemic years than in pre-pandemic years. |
| Dapper 2022 | Germany | Ecological | Analyzed the impact of infection control measures implemented during COVID-19 on infectious diseases at a University Hospital. | June 2019 - June 2021 | Community and hospital | No significant changes were detected in the prevalence of susceptible and drug-resistant bacterial pathogens, although the consumption of hand disinfectants and protective equipment increased. |
| de Carvalho Hessel Dias 2022 | Brazil | Ecological | Analyzed the carbapenem-resistant Gram-negative bacteria incidence density trend and AMU in 99 critical care facilities. | January 2019 - December 2020 | Hospital | CRA increased in 2020 and had a strong positive correlation with the incidence density of COVID-19. Polymyxin consumption also increased. |
| Dutta 2022 | India | Ecological | Compared the hospital-based epidemiology of neonatal sepsis before, during and after coronavirus disease lockdowns. | March 2019 - September 2020 | Hospital | There was a higher proportion of MDR/extreme drug resistance/pan drug resistance sepsis before and after lockdown than during lockdown. |
| Endo 2022 | Japan | Ecological | Assessed the temporal changes in AMR-related metrics before and after the start of the COVID-19 pandemic. | January 2019 - January 2021 | Hospital | The incidence of resistant *S. aureus* and *E. coli* did not show a major temporal change after the start of the COVID-19 pandemic. The incidence of resistant *S. pneumoniae* decreased from April 2020 onward. AMU showed a weak increasing trend, while the use of hand sanitizer increased. |
| Freire 2023 | Brazil | Interrupted Time Series | Analyzed HAI rates and antimicrobial consumption before and during the COVID-19 pandemic. | 2017-2020 | Hospital | HAI increased during COVID-19. The microorganisms’ susceptibility profile did not change, but there was a disproportionate increase in large-spectrum antimicrobial drug use. |
| Fukushige 2022 | Taiwan | Ecological | Investigated the burden and patient characteristics of hospital-associated VRE infections when multiple preventive measures for COVID-19 were taken. | 2018 - 2020 | Hospital | The incidence density of both VRE HAIs and VRE hospital-associated bloodstream infections did not change significantly despite universal mask-wearing and increased consumption of 75% alcohol in 2020. |
| Gaspari 2021 | Italy | Interrupted Time Series | Investigated whether precautions adopted during the COVID-19 pandemic influenced the spreading and MDR of pathogens among ICU patients during the COVID-19 period. | June 2019 - February 2021 | Hospital | These findings suggest that a robust adherence to hygiene measures with human contact restrictions in a COVID-19 free ICU might also restrain the transmission of pathogens. Overall isolates during pandemic less frequently exhibited MDR. |
| Gisselø 2022 | Denmark | Ecological | Investigated change in resistance in VRE *E. faecium* at Copenhagen University Hospital in Denmark. | 2014 - 2020 | Hospital | When comparing the first 5 months of the COVID-19 pandemic with the corresponding period in 2019, there was a 10-fold decrease in VRE *E. faecium* outbreak patients, and the median outbreak duration decreased. |
| Guven 2021 | Turkey | Ecological | Evaluated the nosocomial infection rates over the first 3 months of COVID-19 compared to the same time frame of the previous year. | 2019 - 2020 | Hospital (oncology ward) | The rate of nosocomial infections caused by MDR bacteria was similar between periods. |
| Hibiya 2022 | Japan | Interrupted time series | Examined the incidence of common infectious diseases in Japan during the COVID-19 pandemic. | 2015 - 2020 | Community and Hospital | CRE showed the same trend over the previous five years. The weekly number of cases of measles, rotavirus, and several infections transmitted by droplet spread was negatively correlated with the weekly number of cases of COVID-19. |
| Hosseini 2023 | United States | Ecological | Examined the potential effects of pandemic-related lifestyle changes on the metabolically relevant small bowel microbiome. | 2019-2021 | Community | There were no significant changes in duodenal microbial alpha diversity in the intra-pandemic vs. pre-pandemic group, but beta diversity was significantly different. Potential disruptor genera were significantly lower during COVID-19. |
| Hurtado 2023 | Columbia | Ecological | Assessed changes in antibiotic resistance of eight of the World Health Organization priority bug-drug combinations and consumption of six antibiotics. | 2018-2021 | Hospital | While resistance significantly decreased for four selected bug-drug combinations, the level of resistance for *Enterococcus faecium* to vancomycin significantly increased. There was no change in resistance for three other bug-drug combinations. Consumption of all antibiotics increased. |
| Imoto 2022 | Japan | Ecological | Investigated the effects of COVID-19 on the use of hand sanitizers, the detection of bacteria from blood cultures, and the dose of antibacterial drugs used for one year before and after COVID-19 admissions began. | April 2019 - March 2021 | Hospital | The use of hand sanitizers increased; the incidence of MSSA and all *S. aureus* detected in blood cultures reduced in all departments. No decrease was observed in the usage of all antibacterial drugs; all antibacterial drugs tended to increase in all departments. |
| Ipek 2022 | Turkey | Ecological | Investigated the change in nosocomial infection rate and hand hygiene compliance. | April 2019 - September 2020 | Hospital | During the pandemic, there were decreased cases of *K.* *pneumoniae* and hand hygiene compliance rates increased. |
| Jani 2021 | India | Ecological | Examined the impact of lockdowns and travel restrictions on changes in antibiotic-resistant strains of bacteria in samples taken from waterways | 2015 - 2020 | Community | Functional profiling using targeted metagenomics to look at change in changes in bacterial populations found a reduction in infection and drug-resistance genes. |
| Jeon 2022 | South Korea | Ecological | Examined the prevalence of MDR bacteria during the COVID-19 pandemic compared to in the pre-pandemic period in four university hospitals. | 2018 - 2021 | Hospital (ICU and wards) | The prevalence of MRSA, VRE, CRE, and CRPA isolated in clinical samples from the ward and VRE and CRE isolated from the ICU were significantly increased. Only CRE in surveillance samples increased in the wards. |
| Kastrin 2023 | Solvenia | Ecological | Aimed to investigate the impact of the COVID-19 pandemic on community antibiotic consumption and the resistance of invasive Streptococcus pneumoniae to penicillin. | 2015-2022 | Community | During the pandemic in 2020 and 2021, the total use of antibiotics for systemic use decreased, while the incidence of invasive pneumococcal diseases in Slovenia had a large decline during the pandemic. |
| Kumar 2021 | India | Ecological | To assess the effect of imprudent consumption of antibiotics during the COVID-19 pandemic on antidrug resistance genes in samples taken from waterways. | 2018 - 2020 | Community | Found a significant increase in resistance of E.coli (by incubating E.coli samples taken from waterways and testing susceptibility to six antibiotics) in 2020 compared to 2018 in ambient water bodies. |
| Lemenand 2021 | France | Interrupted time series | Compared ESBL*-E.coli* rates of patients in primary care and nursing home residents before and after the lockdown. | January 2019 - December 2020 | Community | In primary care, percent of *E. coli* isolates producing reduced during the pandemic. |
| Lin 2023 | Taiwan | Ecological | Examined whether obligatory facial masking and reduced health-care capacity because of COVID-19 influenced TB transmission. | 2010-2021 | Community | In Taiwan, TB incidence (and MDR-TB incidence) declined gradually from 2010 to 2021 even during the COVID19 pandemic. TB mortality was the same during the pandemic. |
| Lo 2020 | Taiwan | Ecological | Investigated the impact of IPAC measures on the incidence rates of HAI and MDRO in a medical centre. | 2018 - 2020 | Hospital | Incidence density of MDRO was significantly lower in 2020. |
| Lopez-Jacome 2022 | Mexico | Ecological | Assessed the changes in antimicrobial resistance among some critical and high-priority microorganisms during the COVID-19 pandemic. | 2019 - 2020 | Hospital | Antimicrobial resistance increased in Mexico during the COVID-19 pandemic. |
| Maczynska 2023 | Poland | Ecological | Analyzed changes in antimicrobial use and change in the drug resistance of Gram-negative Pseudomonas aeruginosa and Acinetobacter baumannii infections. | 2017-2022 | Hospital | The number of multi resistant strains of A. baumannii increased; related to increased use, of broad-spectrum antibiotics, mainly penicillins, third-generation cephalosporins and carbapenems. |
| Mannathoko 2022 | Botswanna | Ecological | Determined the prevalence of ESCrE and CRE colonization in hospitals, outpatient clinics, and community settings to evaluate the changes in colonization prevalence during the pandemic. | 2020 | Community and Hospital | For both ESCrE and CRE, there were significant decreases in colonization prevalence after a two-month countrywide lockdown to address the COVID-19 pandemic. |
| Meschiari 2022 | Italy | Interrupted time series | Evaluated the impact of COVID-19 on AMR in the University Hospital. | 2015 - 2021 | Hospital | Found a significant increase only in the level of BSIs due to CRPA. MRSA had a non-significant increase in resistance. |
| Meyer Sauteur 2022 | Global | Ecological | Investigated global *M. pneumoniae* incidence after implementation of IPAC measures against COVID-19 in 21 countries. | April 2020 - March 2021 | Community | In all countries*, M. pneumoniae* incidence by direct test methods declined significantly during the pandemic. A decrease in Macrolide-resistant *M. pneumoniae* (MRMp) rates was also observed. |
| Micozzi 2021 | Italy | Ecological | Evaluated the potential effects of IPAC measures against COVID-19 on KPC-KP transmission in Italy. | November 2019 - August 2020 | Hospital | Percent of hospitalized patients that were KPC-KP positive decreased during the pandemic. |
| Mughini-Gras 2021 | Netherlands | Ecological | This study assessed the impact of COVID-19 pandemic public health measures on human salmonellosis. | 2016-2021 | Community | Salmonellosis incidence decreased significantly after March 2020; The decrease was strongest among travel-related cases. |
| Ochoa-Hein 2021 | Mexico | Ecological | HAI rates were compared before and the COVID-19 hospital surge capacity response. | 2019 - 2020 | Hospital | MRSA, CPE, ESBL producers, ampicillinase C (AmpC) producers and CRE showed no significant changes while MDR *P. aeruginosa* showed a significant reduction between these two periods. |
| Onal 2023 | Turkey | Ecological | Aimed to evaluate the effects of the COVID-19 on healthcare-associated infections, antibiotic resistance, and consumption rates in intensive care units. | 2018-2021 | Hospital | BSI incidence rates were significantly increased in all ICUs during the COVID-19 pandemic. In addition, AMU increased in all ICUs after the start of the COVID-19 pandemic. |
| Pascale 2022 | Italy | Interrupted time series | Assessed the incidence of colonization and infection with CPE and carbapenem-resistant *Acinetobacter* before-and-after the pandemic. | 2019 - 2020 | Hospital | Found no difference in the IRRs of colonization and infection with CPE during the pre-COVID-19 period and the COVID-19 period, whereas the incidence rate ratio (IRR) of CR-Ab increased significantly during the COVID-19 period. |
| Pereira 2023 | Brazil | Ecological | Evaluate the impact of the first year of the COVID-19 pandemic on antibiotic dispensation and resistance rates in three hospitals. | 2018 to 2021 | Hospital | Reduced antibiotic dispensation occurred except for azithromycin dispensation which increased in all hospitals. Macrolide- resistant bacterial isolates rose. |
| Petrakis 2023 | Greece | Ecological | Evaluated the incidence of antimicrobial resistance of bloodstream infections before and during the COVID-19 pandemic. | 2018 to 2022 | Hospital | An increasing trend was reported compared to the pre-pandemic period in the incidence of resistant Gram-negative bacteria, particularly in ICUs. Infectious disease consultations decreased, and telephone consultations increased. |
| Russotto 2023 | Italy | Ecological | This study evaluated the potential COVID-19 pandemic impact on hand hygiene practices and rate of healthcare-associated infections. | 2017 to 2021 | Hospital | A significant increase in alcohol hand rub consumption was seen. A significant decrease in MRSA and decreasing CRE rates was also found. |
| Santos 2022 | United States | Ecological | Measured facility-wide antimicrobial use/antimicrobial resistance ratios during the pandemic. | 2019 - 2020 | Hospital | Some antibiotics like vancomycin, linezolid, ceftolozane–tazobactam, and colistin did not differ significantly in use between two time periods. Significant decreases were seen in meropenem and daptomycin use and increases in ceftazidime–avibactam. ESBL-Enterobacterales events significantly increased during COVID-19, along with CRE and VRE events. No differences were observed in the median monthly number of events for CNA, MRSA, and MDR *P. aeruginosa.* |
| Sasaki 2022 | Japan | Ecological | Assessed antimicrobial consumption and MDRO incidence in a small hospital during and before the pandemic. | 2018 - 2022 | Hospital | Found no change in the incidence of MRSA but found an increase in the ESBL-E incidence during the pandemic. The consumption of intravenous antimicrobials increased significantly. |
| Shbaklo 2022 | Italy | Ecological | Examined incidence of MDR HAIs and antibiotic consumption during the three waves of COVID-19 compared to before. | 2019 - 2021 | Hospital | Demonstrated an increase in MDR infections. The use of fourth- and fifth-generation cephalosporins and piperacillin–tazobactam increased at the beginning of the COVID period while fluoroquinolone use decreased. |
| Soto Hernandez 2023 | Mexico | Ecological | Evaluated the impact of the COVID-19 pandemic at a neurosurgical reference centre. | 2019-2022 | Hospital | In 2020 the total number of surgeries was reduced. The rate of neurosurgical infections increased however no significant differences were found for patterns of resistance to antibiotics. |
| Tang 2022 | Taiwan | Ecological | Compared the number of cases of airborne/droplet-transmitted notifiable infectious diseases before and during the pandemic. | 2018 - 2021 | Hospital | Most pathogens decreased during the pandemic including MDR-TB. |
| Tedeschi 2023 | Italy | Ecological | The aim of this study was to assess antibiotic consumption and antibiotic resistance before and during the COVID-19 pandemic. | 2019-2020 | Community | Overall antibiotic consumption decreased and strains of *Enterobacterales* showed increasing susceptibility to amoxicillin/clavulanate from isolates from primary and long-term care. |
| Teixeira 2022 | Portugal | Ecological | Compared the rate of postoperative infection and drug-resistant organisms before and during the COVID-19 pandemic. | 2018 - 2020 | Hospital | Postoperative infection rates were not significantly reduced during the COVID-19 pandemic, despite the adoption of enhanced infection preventive measures. There was, however, a decrease in the rate of DROs during this period. |
| Tham 2022 | Australia | Retrospective cohort | Determined the effect of the COVID-19 pandemic IPAC measures on the incidence of HAI in surgical patients. | April 2019 - June 2020 | Hospital (surgical) | There were no major changes in the types of microorganisms involved in HAI across the two study periods. |
| Ullrich 2021 | Germany | Interrupted time series | Assessed the impact of the pandemic and COVID-19 IPAC measures on infectious diseases. | 2020 | Community and Hospital | The number of cases decreased for respiratory diseases, gastro-intestinal diseases and imported vector-borne diseases. Hospital associated infections like MRSA and sexually transmitted, and blood-borne diseases also decreased. |
| Vyazovaya 2022 | Russia | Ecological | Examined how counteracting factors imposed by the pandemic could influence changes in the local *M. tuberculosis* population. | 2019- 2021 | Community | No change was observed in the *M. tuberculosis* population structure during the Covid-19 pandemic but there was a decrease of the Beijing genotype which are mostly MDR and an increase in the proportion and diversity of the non-Beijing isolates. |
| Wee 2021 | Singapore | Ecological | Evaluated the impact of a multimodal IPAC COVID-19 strategy on the rates of HAI across a large health care campus in Singapore. | 2018 - 2020 | Hospital | No increase in CP-CRE acquisition, and rates of other HAIs were stable. Hospital-wide MRSA acquisition rates declined significantly during the pandemic. |
| Wong 2023 | Hong Kong | Ecological | Examined the epidemiology of MDRO and antibiotic use before and during the COVID-19 pandemic. | 2016-2022 | Hospital | Found a significant increase in AMU and increasing trends of CRA infections, while there was no significant increase in MRSA and ESBL-producing Enterobacterales infections. Increased compliance of hand hygiene per year. |
| Yang 2021 | China | Ecological | MRSA detection rates in medical institutions and exposure rates to environmental disinfectants before and during the pandemic. | 2016 - 2020 | Hospital | The MRSA detection rate increased with elevated concentration and frequency of disinfection. |
| Zaveri 2021 | India | Ecological | Monitored change in AMR among pathogens found in air samples, and surface swabs at three tertiary care hospitals before and during the pandemic. | 2017 - 2020 | Hospital | Carbapenem-resistant genes decreased pre and post pandemic. The prevalence of pathogenic (*Klebsiella spp., E. coli,* and *Pseudomonas spp.)* and non-pathogenic (*S. aureus* and *Bacillus spp.*) strains decreased. |
| Zhu 2022 | United Kingdom | Ecological | Examined community- and hospital-associated BSIs across 2 epidemic waves. | 2020 - 2021 | Hospital and community | Hospital-associated BSI caused by MRSA had the largest increase among all causative pathogens in both COVID-19 and non–COVID-19 patients, compared to pre–COVID-19 figures. The overall rates of community-associated BSI and MRSA were lower than the pre–COVID-19 level but peaked following lockdown easing in May 2020. |
| Zhu 2022 | China | Ecological | Measured distribution and drug resistance of bacterial pathogens associated with lower respiratory tract infection and impact of COVID-19 measures. | 2011 - 2020 | Community | The resistance of *E. coli* and *K. pneumoniae* to third generation cephalosporins was decreasing. Detection rate of MRSA showed an increasing trend with the increase of oxacillin resistance. |
| Zondag 2023 | Netherlands | Ecological | This study investigated the effect of COVID-19 IPAC measures on the genotypic and phenotypic distribution of Neisseria gonorrhoeae isolates. | 2020 | Community | Phenotypic data showed an increase in low- level azithromycin resistance and ceftriaxone susceptibility during the lockdown, and this remained after the study period. |
| Zuglian 2022 | Italy | Ecological | Compared the prevalence and the antibiotic profile of bacterial and fungal species of patients before and during COVID-19. | 2019 - 2020 | Hospital (ICU) | There was a statistically significant increase in resistance of *Pseudomonas spp.* to carbapenems and piperacillin/tazobactam and *Enterobacterales spp.* for piperacillin/tazobactam. |

Multidrug-resistant (MDR), multidrug-resistant organisms (MDRO), hospital-associated infections (HAIs), bloodstream infection (BSI), community-associated infections (CAIs), Carbapenemase Producing Carbapenem-Resistant Enterobacteriaceae (CP-CRE), central-line–associated bloodstream infections (CLABSIs), catheter-associated urinary tract infections (CAUTIs), bloodstream infections (BSI), Vancomycin-resistant *Enterococcus* (VRE), methicillin-resistant *Staphylococcus aureus* (MRSA), methicillin-susceptible *Staphylococcus aureus* (MSSA), carbapenemase-producing *Klebsiella pneumoniae* (KPC-KP), extended-spectrum beta-lactamase (ESBL), carbapenem-resistant *Enterobacteriaceae* (CRE), carbapenem-non-susceptible *Acinetobacter* (CNA), extended-spectrum cephalosporin-resistant *Enterobacterales* (ESCrE), carbapenem-resistant *Acinetobacter baumannii* (CRA), carbapenem-resistant *Pseudomonas aeruginosa* (CRPA), fluconazole-resistant *Candida parapsilosis* (FRCP), non-pharmaceutical interventions (NPI), infection prevention and control (IPAC)

*Table S2. PROGRESS-Plus factors for each study*

| Study author, year | PROGRESS | | | | | | | | PLUS | | |
| --- | --- | --- | --- | --- | --- | --- | --- | --- | --- | --- | --- |
|  | **Place of residence** | **Race, ethnicity, culture, or language** | **Occupation** | **Gender or sex** | **Religion** | **Education** | **Socio-economic status** | **Social capital** | **Personal characteristics associated with discrimination (e.g., age, disability)** | **Features of relationships (e.g., smoking parents, excluded from school)** | **Time-dependent relationships (e.g., leaving the hospital, respite care, other instances where a person may be temporarily at a disadvantage)** |
| Alao 2022 | no | no | no | yes | no | no | no | no | yes | no | no |
| Aldeyab 2023 | no | no | no | no | no | no | no | no | no | no | no |
| Allel 2023 | no | no | no | no | no | no | no | no | no | no | yes |
| Bauer 2022 | yes | no | no | yes | no | no | no | no | yes | no | yes |
| Bentivegna 2021 | no | no | no | no | no | no | no | no | no | no | no |
| Bork 2020 | no | no | no | no | no | no | no | no | no | no | no |
| Bussolati 2022 | no | no | no | yes | no | no | no | no | yes | no | yes |
| Chamieh 2021 | no | no | no | no | no | no | no | no | no | no | no |
| Chang 2023 | no | no | no | no | no | no | no | no | no | no | no |
| Chen 2021 | no | no | no | yes | no | no | no | no | yes | no | yes |
| Cheng 2022 | no | no | no | no | no | no | no | no | no | no | no |
| Dapper 2022 | no | no | no | no | no | no | no | no | no | no | no |
| de Carvalho Hessel Dias 2022 | no | no | no | no | no | no | no | no | no | no | no |
| Dutta 2022 | no | no | no | yes | no | no | no | no | yes | no | yes |
| Endo 2023 | no | no | no | no | no | no | no | no | no | no | no |
| Freire 2023 | no | no | no | no | no | no | no | no | no | no | no |
| Fukushige 2022 | no | no | no | yes | no | no | no | no | yes | no | yes |
| Gaspari 2021 | no | no | no | yes | no | no | no | no | yes | no | yes |
| Gisselo 2022 | no | no | no | no | no | no | no | no | no | no | no |
| Guven 2021 | no | no | no | no | no | no | no | no | no | no | no |
| Hibiya 2022 | no | no | no | no | no | no | no | no | no | no | no |
| Hosseini 2023 | no | no | no | yes | no | no | no | no | yes | no | yes |
| Hurtado 2023 | no | no | no | no | no | no | no | no | no | no | no |
| Imoto 2022 | no | no | no | no | no | no | no | no | no | no | no |
| Ipek 2022 | no | no | no | yes | no | no | no | no | yes | no | yes |
| Jani 2021 | no | no | no | no | no | no | no | no | no | no | no |
| Jeon 2022 | no | no | no | no | no | no | no | no | no | no | no |
| Kastrin 2023 | no | no | no | yes | no | no | no | no | yes | no | no |
| Kumar 2021 | no | no | no | no | no | no | no | no | no | no | no |
| Lemenand 2021 | yes | no | no | yes | no | no | no | no | yes | no | no |
| Lo 2020 | no | no | no | no | no | no | no | no | no | no | no |
| Lopez-Jacome 2022 | no | no | no | no | no | no | no | no | no | no | no |
| Maczynska 2023 | no | no | no | no | no | no | no | no | no | no | no |
| Mannathoko 2022 | no | no | no | yes | no | no | no | no | yes | no | yes |
| Meschiari 2022 | no | no | no | no | no | no | no | no | no | no | no |
| Meyer Sauteur 2022 | yes | no | no | yes | no | no | no | no | yes | no | no |
| Micozzi 2021 | no | no | no | yes | no | no | no | no | yes | no | yes |
| Mughini-Gras 2021 | no | no | no | yes | no | no | no | no | yes | no | yes |
| Ochoa-Hein 2021 | no | no | no | yes | no | no | no | no | yes | no | yes |
| Onal 2023 | no | no | no | no | no | no | no | no | yes | no | yes |
| Pascale 2022 | no | no | no | yes | no | no | no | no | yes | no | yes |
| Pereira 2023 | no | no | no | no | no | no | no | no | no | no | no |
| Petrakis 2023 | no | no | no | yes | no | no | no | no | yes | no | yes |
| Russotto 2023 | no | no | no | no | no | no | no | no | no | no | no |
| Santos 2022 | no | no | no | no | no | no | no | no | no | no | no |
| Sasaki 2022 | no | no | no | no | no | no | no | no | no | no | no |
| Shbaklo 2022 | no | no | no | no | no | no | no | no | no | no | no |
| Soto Hernandez 2023 | no | no | no | no | no | no | no | no | yes | no | yes |
| Tang 2022 | no | no | no | no | no | no | no | no | no | no | no |
| Teixeira 2022 | no | no | no | yes | no | no | no | no | yes | no | yes |
| Tham 2022 | no | no | no | yes | no | no | no | no | yes | no | yes |
| Ullrich 2021 | yes | no | no | yes | no | no | no | no | yes | no | no |
| Vyazovaya 2022 | no | no | no | no | no | no | no | no | no | no | no |
| Wee 2021 | no | no | no | no | no | no | no | no | no | no | no |
| Wong 2023 | no | no | no | yes | no | no | no | no | yes | no | yes |
| Yang 2021 | no | no | no | no | no | no | no | no | no | no | no |
| Zaveri 2021 | no | no | no | no | no | no | no | no | no | no | no |
| Zhu 2022 (China) | no | no | no | yes | no | no | no | no | yes | no | no |
| Zhu 2022 (UK) | no | yes | no | yes | no | no | no | no | yes | no | yes |
| Zondag 2023 | no | no | no | yes | no | no | no | no | yes | no | no |
| Zuglian 2022 | no | no | no | yes | no | no | no | no | yes | no | yes |


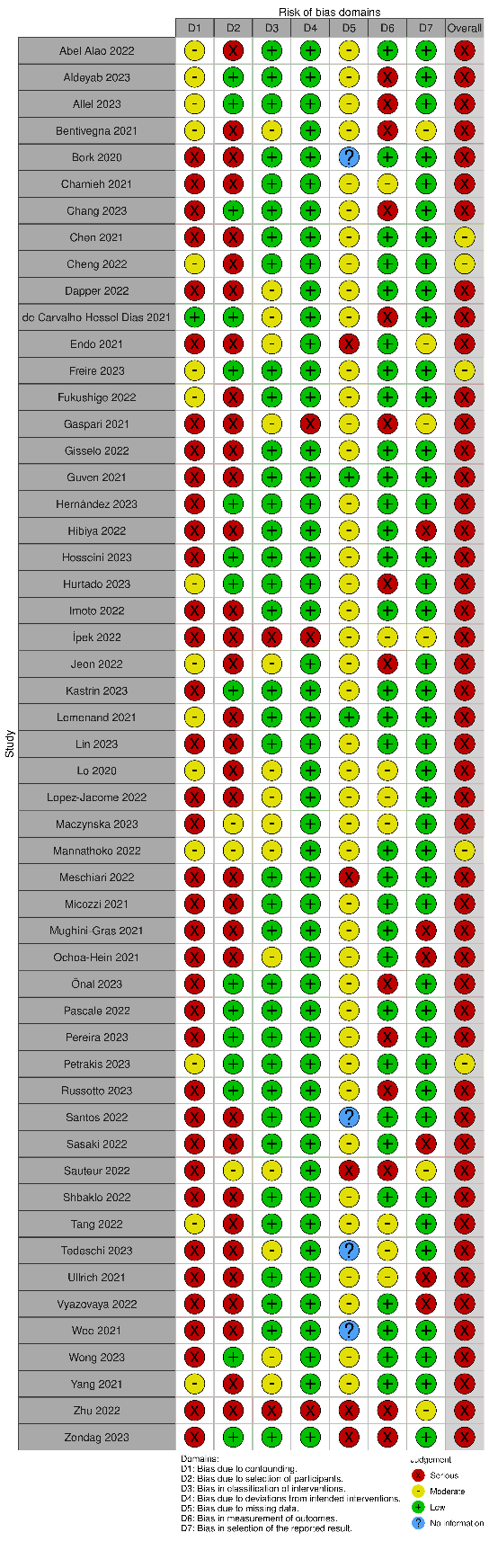


*Figure S1. Summary of risk of bias and applicability concerns evaluated using the ROBINS-I quality assessment tool for non-randomized studies.*

| **Study ID** | **Risk of bias**  **(NOS)** | | | |
| --- | --- | --- | --- | --- |
|  | **S** | **C** | **O** | **F** |
|  |  |  |  |  |
| Bauer 2022 | ** | ** | * | 5/9 stars (Moderate Risk of Bias) |
| Bussolati 2022 | ** | * | * | 5/9 stars (Moderate Risk of Bias) |
| Dutta 2022 | ** | * | * | 4/9 stars (Moderate Risk of Bias) |
| Teixeira 2021 | ** | ** | * | 5/9 stars (Moderate Risk of Bias) |
| Tham 2022 | *** | ** | * | 6/9 stars (Moderate Risk of Bias) |

S = selection; C = comparability; O = outcome; F = final overall rating

*Figure S2. Summary of risk of bias and applicability concerns evaluated using the Newcastle Ottawa Scale (NOS) for cohort studies*.

| **Study ID** | **Criterion 1:**  **Risk of**  **Confounding**  **Biases** | **Criterion 2:**  **Risk of Post-**  **Intervention/**  **Exposure Selection**  **Biases** | **Criterion 3:**  **Risk of Misclassified**  **Comparison Biases** | **Criterion 4:**  **Risk of**  **Performance**  **Biases** | **Criterion 5:**  **Risk of Detection**  **Biases** | **Criterion 6:**  **Risk of Outcome**  **Reporting Biases** | **Criterion 7:**  **Risk of Outcome**  **Assessment Biases** | **Overall risk of bias** |
| --- | --- | --- | --- | --- | --- | --- | --- | --- |
| Jani 2021 | High risk of bias | Medium risk of bias | Low risk of bias | Not applicable | Low risk of bias | Medium risk of bias | Medium risk of bias | High |
| Kumar 2021 | High risk of bias | Low risk of bias | Low risk of bias | Not applicable | Low risk of bias | Medium risk of bias | Medium risk of bias | High |
| Zaveri 2021 | High risk of bias | Low risk of bias | Low risk of bias | Not applicable | Low risk of bias | Medium risk of bias | Medium risk of bias | High |

*Figure S3. Summary of risk of bias concerns evaluated using the Collaboration for Environmental Evidence Critical Appraisal Tool for environmental sampling studies.*

**APPENDIX 2**

*Search Strategy*

Covid-19 – Antimicrobial Resistance

Final Strategies

2022 Dec 19

Ovid Multifile

Database: Embase Classic+Embase <1947 to 2022 December 16>, Ovid MEDLINE(R) ALL <1946 to December 16, 2022>, EBM Reviews - Cochrane Central Register of Controlled Trials <November 2022>, EBM Reviews - Cochrane Database of Systematic Reviews <2005 to December 14, 2022>

Search Strategy:

--------------------------------------------------------------------------------

1 COVID-19/ (272610)

2 SARS-CoV-2/ (169494)

3 Coronavirus/ (14858)

4 Betacoronavirus/ (39932)

5 Coronavirus Infections/ (56651)

6 (COVID-19 or COVID19).tw,kw,kf. (601297)

7 ((coronavirus* or corona virus*) and (hubei or wuhan or beijing or shanghai)).tw,kw,kf. (13884)

8 (wuhan adj5 virus*).tw,kw,kf. (833)

9 (2019-nCoV or 19nCoV or 2019nCoV).tw,kw,kf. (4565)

10 (nCoV or n-CoV or "CoV 2" or CoV2).tw,kw,kf. (234065)

11 (SARS-CoV-2 or SARS-CoV2 or SARSCoV-2 or SARSCoV2 or SARS2 or SARS-2 or severe acute respiratory syndrome coronavirus 2).tw,kw,kf. (237842)

12 (2019-novel CoV or Sars-coronavirus2 or Sars-coronavirus-2 or SARS-like coronavirus* or ((novel or new or nouveau) adj2 (CoV or nCoV or covid or coronavirus* or corona virus or Pandemi*2)) or (coronavirus* and pneumonia)).tw,kw,kf. (59479)

13 (novel coronavirus* or novel corona virus* or novel CoV).tw,kw,kf. (27082)

14 ((coronavirus* or corona virus*) adj2 "2019").tw,kw,kf. (120389)

15 ((coronavirus* or corona virus*) adj2 "19").tw,kw,kf. (17858)

16 ("coronavirus 2" or "corona virus 2").tw,kw,kf. (65337)

17 (OC43 or NL63 or 229E or HKU1 or HCoV* or Sars-coronavirus*).tw,kw,kf. (10003)

18 COVID-19.rx,px,ox. or severe acute respiratory syndrome coronavirus 2.os. (19107)

19 (coronavirus* or corona virus*).ti,kw,kf. (110557)

20 COVID.ti,kw,kf. (517646)

21 ("B.1.1.7" or "B.1.351" or "B.1.617" or "B.1.427" or "B.1.429").tw,kw,kf,rx,px,ox. (3546)

22 ("BA.1" or "BA.2" or "BA.3" or "BA.4" or "BA.5" or "BA.2.75" or "BA.4.6" or "BA.2.3.20" or "XBB").tw,kw,kf,rx,px,ox. (11090)

23 ("P.1" and (Brazil* or variant?)).tw,kw,kf,rx,px,ox. (4706)

24 (((alpha or beta or delta or eta or gamma or iota or kappa or lambda or omicron or zeta) adj3 variant?) and (coronavirus* or corona virus* or covid*)).tw,kw,kf. (9714)

25 or/1-24 [COVID-19] (723180)

26 exp Drug Resistance, Microbial/ (393502)

27 ((antibiotic? or anti-biotic? or abx or antibacterial? or anti-bacterial? or antifungal? or anti-fungal? or antimicrobial? or anti-microbial? or antiviral? or anti-viral? or bacterial? or microbial?) adj5 (resistan* or nonsusceptib* or non-susceptib*)).tw,kw,kf. (324394)

28 (AMR adj10 resistan*).tw,kw,kf. (9794)

29 ((multidrug? or multi-drug? or multiple drug?) adj5 (resistan* or nonsusceptib* or non-susceptib*)).tw,kw,kf. (181848)

30 ((betalactam* or beta-lactam* or b-lactam* or blactam*) adj5 (resistan* or nonsusceptib* or non-susceptib*)).tw,kw,kf. (28274)

31 (cephalosporin* adj5 (resistan* or nonsusceptib* or non-susceptib*)).tw,kw,kf. (11879)

32 ((penicillin* or ampicillin* or methicillin*) adj5 (resistan* or nonsusceptib* or non-susceptib*)).tw,kw,kf. (125933)

33 (carbapenem* adj5 (resistan* or nonsusceptib* or non-susceptib*)).tw,kw,kf. (27808)

34 (chloramphenicol* adj5 (resistan* or nonsusceptib* or non-susceptib*)).tw,kw,kf. (10755)

35 (daptomycin* adj5 (resistan* or nonsusceptib* or non-susceptib*)).tw,kw,kf. (2117)

36 ((fluoroquinolone* or ciprofloxacin* or enoxacin* or enrofloxacin* or fleroxacin* or gatifloxacin* or gemifloxacin* or levofloxacin* or moxifloxacin* or norfloxacin* or ofloxacin* or pefloxacin*) adj5 (resistan* or nonsusceptib* or non-susceptib*)).tw,kw,kf. (34276)

37 ((macrolide* or ado-trastuzumab emtansine* or everolimus* or fidaxomicin* or lucensomycin* or maytansine* or mepartricin* or miocamycin* or natamycin or nystatin* or oleandomycin* or oligomycin* or rutamycin* or sirolimus* or tacrolimus* or troleandomycin* or tylosin*) adj5 (resistan* or nonsusceptib* or non-susceptib*)).tw,kw,kf. (13936)

38 ((erythromycin* or azithromycin* or clarithromycin* or ketolide* or roxithromycin*) adj5 (resistan* or nonsusceptib* or non-susceptib*)).tw,kw,kf. (23588)

39 (kanamycin* adj5 (resistan* or nonsusceptib* or non-susceptib*)).tw,kw,kf. (7947)

40 ((polymyxin* or poly-myxin* or colistin*) adj5 (resistan* or nonsusceptib* or non-susceptib*)).tw,kw,kf. (10679)

41 (rifampicin* adj5 (resistan* or nonsusceptib* or non-susceptib*)).tw,kw,kf. (10011)

42 (tetracycline* adj5 (resistan* or nonsusceptib* or non-susceptib*)).tw,kw,kf. (22980)

43 (trimethoprim* adj5 (resistan* or nonsusceptib* or non-susceptib*)).tw,kw,kf. (8581)

44 (vancomycin* adj5 (resistan* or nonsusceptib* or non-susceptib*)).tw,kw,kf. (26464)

45 Antimicrobial Stewardship/ (12229)

46 ((antibiotic? or anti-biotic? or abx or antibacterial? or anti-bacterial? or antifungal? or anti-fungal? or antimicrobial? or anti-microbial? or antiviral? or anti-viral? or bacterial? or microbial?) adj5 (custodian* or guardian* or oversee* or oversight* or safeguard* or safe guard* or steward* or watchdog? or watch dog?)).tw,kw,kf. (24623)

47 or/26-46 [AMR] (765724)

48 25 and 47 [COVID-19 - AMR] (4875)

49 limit 48 to yr="2020-current" [DATE LIMIT] (4426)

50 49 use medall [MEDLINE RECORDS] (1872)

51 coronavirus disease 2019/ (487541)

52 severe acute respiratory syndrome coronavirus 2/ (224399)

53 Coronavirinae/ (6402)

54 Betacoronavirus/ (39932)

55 coronavirus infection/ (57539)

56 (COVID-19 or COVID19).tw,kw,kf. (601297)

57 ((coronavirus* or corona virus*) and (hubei or wuhan or beijing or shanghai)).tw,kw,kf. (13884)

58 (wuhan adj5 virus*).tw,kw,kf. (833)

59 (2019-nCoV or 19nCoV or 2019nCoV).tw,kw,kf. (4565)

60 (nCoV or n-CoV or "CoV 2" or CoV2).tw,kw,kf. (234065)

61 (SARS-CoV-2 or SARS-CoV2 or SARSCoV-2 or SARSCoV2 or SARS2 or SARS-2 or severe acute respiratory syndrome coronavirus 2).tw,kw,kf. (237842)

62 (2019-novel CoV or Sars-coronavirus2 or Sars-coronavirus-2 or SARS-like coronavirus* or ((novel or new or nouveau) adj2 (CoV or nCoV or covid or coronavirus* or corona virus or Pandemi*2)) or (coronavirus* and pneumonia)).tw,kw,kf. (59479)

63 (novel coronavirus* or novel corona virus* or novel CoV).tw,kw,kf. (27082)

64 ((coronavirus* or corona virus*) adj2 "2019").tw,kw,kf. (120389)

65 ((coronavirus* or corona virus*) adj2 "19").tw,kw,kf. (17858)

66 ("coronavirus 2" or "corona virus 2").tw,kw,kf. (65337)

67 (OC43 or NL63 or 229E or HKU1 or HCoV* or Sars-coronavirus*).tw,kw,kf. (10003)

68 (coronavirus* or corona virus*).ti,kw,kf. (110557)

69 COVID.ti,kw,kf. (517646)

70 ("B.1.1.7" or "B.1.351" or "B.1.617" or "B.1.427" or "B.1.429").tw,kw,kf. (3503)

71 ("BA.1" or "BA.2" or "BA.3" or "BA.4" or "BA.5" or "BA.2.75" or "BA.4.6" or "BA.2.3.20" or "XBB").tw,kw,kf. (11036)

72 ("P.1" and (Brazil* or variant?)).tw,kw,kf. (4669)

73 (((alpha or beta or delta or eta or gamma or iota or kappa or lambda or omicron or zeta) adj3 variant?) and (coronavirus* or corona virus* or covid*)).tw,kw,kf. (9714)

74 or/51-73 [COVID-19] (739570)

75 exp antibiotic resistance/ (393502)

76 antifungal resistance/ (6177)

77 antiviral resistance/ (9688)

78 ((antibiotic? or antibiotic? or abx or antibacterial? or anti-bacterial? or antifungal? or anti-fungal? or antimicrobial? or anti-microbial? or antiviral? or anti-viral? or bacterial? or microbial?) adj5 (resistan* or nonsusceptib* or non-susceptib*)).tw,kw,kf. (324368)

79 (AMR adj10 resistan*).tw,kw,kf. (9794)

80 ((multidrug? or multi-drug? or multiple drug?) adj5 (resistan* or nonsusceptib* or non-susceptib*)).tw,kw,kf. (181848)

81 ((betalactam* or beta-lactam* or b-lactam* or blactam*) adj5 (resistan* or nonsusceptib* or non-susceptib*)).tw,kw,kf. (28274)

82 (cephalosporin* adj5 (resistan* or nonsusceptib* or non-susceptib*)).tw,kw,kf. (11879)

83 ((penicillin* or ampicillin* or methicillin*) adj5 (resistan* or nonsusceptib* or non-susceptib*)).tw,kw,kf. (125933)

84 (carbapenem* adj5 (resistan* or nonsusceptib* or non-susceptib*)).tw,kw,kf. (27808)

85 (chloramphenicol* adj5 (resistan* or nonsusceptib* or non-susceptib*)).tw,kw,kf. (10755)

86 (daptomycin* adj5 (resistan* or nonsusceptib* or non-susceptib*)).tw,kw,kf. (2117)

87 ((fluoroquinolone* or ciprofloxacin* or enoxacin* or enrofloxacin* or fleroxacin* or gatifloxacin* or gemifloxacin* or levofloxacin* or moxifloxacin* or norfloxacin* or ofloxacin* or pefloxacin*) adj5 (resistan* or nonsusceptib* or non-susceptib*)).tw,kw,kf. (34276)

88 ((macrolide* or ado-trastuzumab emtansine* or everolimus* or fidaxomicin* or lucensomycin* or maytansine* or mepartricin* or miocamycin* or natamycin or nystatin* or oleandomycin* or oligomycin* or rutamycin* or sirolimus* or tacrolimus* or troleandomycin* or tylosin*) adj5 (resistan* or nonsusceptib* or non-susceptib*)).tw,kw,kf. (13936)

89 ((erythromycin* or azithromycin* or clarithromycin* or ketolide* or roxithromycin*) adj5 (resistan* or nonsusceptib* or non-susceptib*)).tw,kw,kf. (23588)

90 (kanamycin* adj5 (resistan* or nonsusceptib* or non-susceptib*)).tw,kw,kf. (7947)

91 ((polymyxin* or poly-myxin* or colistin*) adj5 (resistan* or nonsusceptib* or non-susceptib*)).tw,kw,kf. (10679)

92 (rifampicin* adj5 (resistan* or nonsusceptib* or non-susceptib*)).tw,kw,kf. (10011)

93 (tetracycline* adj5 (resistan* or nonsusceptib* or non-susceptib*)).tw,kw,kf. (22980)

94 (trimethoprim* adj5 (resistan* or nonsusceptib* or non-susceptib*)).tw,kw,kf. (8581)

95 (vancomycin* adj5 (resistan* or nonsusceptib* or non-susceptib*)).tw,kw,kf. (26464)

96 antimicrobial stewardship.mp. [mp=ti, ab, hw, tn, ot, dm, mf, dv, kf, fx, dq, bt, nm, ox, px, rx, ui, sy, sh, kw, tx, ct] (20611)

97 ((antibiotic? or antibiotic? or abx or antibacterial? or anti-bacterial? or antifungal? or anti-fungal? or antimicrobial? or anti-microbial? or antiviral? or anti-viral? or bacterial? or microbial?) adj5 (custodian* or guardian* or oversee* or oversight* or safeguard* or safe guard* or steward* or watchdog? or watch dog?)).tw,kw,kf. (24622)

98 or/75-97 [AMR] (776935)

99 74 and 98 [COVID-19 - AMR] (5312)

100 limit 99 to yr="2020-current" [DATE LIMIT] (4869)

101 100 use emczd [EMBASE RECORDS] (2960)

102 COVID-19/ (272610)

103 SARS-CoV-2/ (169494)

104 Coronavirus/ (14858)

105 Betacoronavirus/ (39932)

106 Coronavirus Infections/ (56651)

107 (COVID-19 or COVID19).ti,ab,kw. (599342)

108 ((coronavirus* or corona virus*) and (hubei or wuhan or beijing or shanghai)).ti,ab,kw. (13709)

109 (wuhan adj5 virus*).ti,ab,kw. (810)

110 (2019-nCoV or 19nCoV or 2019nCoV).ti,ab,kw. (4313)

111 (nCoV or n-CoV or "CoV 2" or CoV2).ti,ab,kw. (206245)

112 (SARS-CoV-2 or SARS-CoV2 or SARSCoV-2 or SARSCoV2 or SARS2 or SARS-2 or severe acute respiratory syndrome coronavirus 2).ti,ab,kw. (235807)

113 (2019-novel CoV or Sars-coronavirus2 or Sars-coronavirus-2 or SARS-like coronavirus* or ((novel or new or nouveau) adj2 (CoV or nCoV or covid or coronavirus* or corona virus or Pandemi*2)) or (coronavirus* and pneumonia)).ti,ab,kw. (57271)

114 (novel coronavirus* or novel corona virus* or novel CoV).ti,ab,kw. (26519)

115 ((coronavirus* or corona virus*) adj2 "2019").ti,ab,kw. (116746)

116 ((coronavirus* or corona virus*) adj2 "19").ti,ab,kw. (16148)

117 ("coronavirus 2" or "corona virus 2").ti,ab,kw. (61868)

118 (OC43 or NL63 or 229E or HKU1 or HCoV* or Sars-coronavirus*).ti,ab,kw. (9923)

119 (coronavirus* or corona virus*).ti,kw. (104607)

120 COVID.ti,kw. (450177)

121 ("B.1.1.7" or "B.1.351" or "B.1.617" or "B.1.427" or "B.1.429").ti,ab,kw. (3475)

122 ("BA.1" or "BA.2" or "BA.3" or "BA.4" or "BA.5" or "BA.2.75" or "BA.4.6" or "BA.2.3.20" or "XBB").ti,ab,kw. (10989)

123 ("P.1" and (Brazil* or variant?)).ti,ab,kw. (4612)

124 (((alpha or beta or delta or eta or gamma or iota or kappa or lambda or omicron or zeta) adj3 variant?) and (coronavirus* or corona virus* or covid*)).ti,ab,kw. (9481)

125 or/102-124 [COVID-19] (721737)

126 exp Drug Resistance, Microbial/ (393502)

127 ((antibiotic? or anti-biotic? or abx or antibacterial? or anti-bacterial? or antifungal? or anti-fungal? or antimicrobial? or anti-microbial? or antiviral? or anti-viral? or bacterial? or microbial?) adj5 (resistan* or nonsusceptib* or non-susceptib*)).ti,ab,kw. (306824)

128 (AMR adj10 resistan*).ti,ab,kw. (9392)

129 ((multidrug? or multi-drug? or multiple drug?) adj5 (resistan* or nonsusceptib* or non-susceptib*)).ti,ab,kw. (175149)

130 ((betalactam* or beta-lactam* or b-lactam* or blactam*) adj5 (resistan* or nonsusceptib* or non-susceptib*)).ti,ab,kw. (27748)

131 (cephalosporin* adj5 (resistan* or nonsusceptib* or non-susceptib*)).ti,ab,kw. (11701)

132 ((penicillin* or ampicillin* or methicillin*) adj5 (resistan* or nonsusceptib* or non-susceptib*)).ti,ab,kw. (124476)

133 (carbapenem* adj5 (resistan* or nonsusceptib* or non-susceptib*)).ti,ab,kw. (26649)

134 (chloramphenicol* adj5 (resistan* or nonsusceptib* or non-susceptib*)).ti,ab,kw. (10698)

135 (daptomycin* adj5 (resistan* or nonsusceptib* or non-susceptib*)).ti,ab,kw. (2077)

136 ((fluoroquinolone* or ciprofloxacin* or enoxacin* or enrofloxacin* or fleroxacin* or gatifloxacin* or gemifloxacin* or levofloxacin* or moxifloxacin* or norfloxacin* or ofloxacin* or pefloxacin*) adj5 (resistan* or nonsusceptib* or non-susceptib*)).ti,ab,kw. (33932)

137 ((macrolide* or ado-trastuzumab emtansine* or everolimus* or fidaxomicin* or lucensomycin* or maytansine* or mepartricin* or miocamycin* or natamycin or nystatin* or oleandomycin* or oligomycin* or rutamycin* or sirolimus* or tacrolimus* or troleandomycin* or tylosin*) adj5 (resistan* or nonsusceptib* or non-susceptib*)).ti,ab,kw. (13739)

138 ((erythromycin* or azithromycin* or clarithromycin* or ketolide* or roxithromycin*) adj5 (resistan* or nonsusceptib* or non-susceptib*)).ti,ab,kw. (23313)

139 (kanamycin* adj5 (resistan* or nonsusceptib* or non-susceptib*)).ti,ab,kw. (7923)

140 ((polymyxin* or poly-myxin* or colistin*) adj5 (resistan* or nonsusceptib* or non-susceptib*)).ti,ab,kw. (10449)

141 (rifampicin* adj5 (resistan* or nonsusceptib* or non-susceptib*)).ti,ab,kw. (9937)

142 (tetracycline* adj5 (resistan* or nonsusceptib* or non-susceptib*)).ti,ab,kw. (22900)

143 (trimethoprim* adj5 (resistan* or nonsusceptib* or non-susceptib*)).ti,ab,kw. (8561)

144 (vancomycin* adj5 (resistan* or nonsusceptib* or non-susceptib*)).ti,ab,kw. (26153)

145 Antimicrobial Stewardship/ (12229)

146 ((antibiotic? or anti-biotic? or abx or antibacterial? or anti-bacterial? or antifungal? or anti-fungal? or antimicrobial? or anti-microbial? or antiviral? or anti-viral? or bacterial? or microbial?) adj5 (custodian* or guardian* or oversee* or oversight* or safeguard* or safe guard* or steward* or watchdog? or watch dog?)).ti,ab,kw. (21963)

147 or/126-146 [AMR] (753702)

148 125 and 147 [COVID-19 - AMR] (4649)

149 -current” (44809373)

150 148 and 149 (4511)

151 limit 148 to yr="2020-current" (4239)

152 150 or 151 (4511) [DATE LIMITS]

153 152 use coch [CDSR RECORDS] (1)

154 152 use cctr [CENTRAL RECORDS] (39)

155 50 or 101 or 153 or 154 [ALL DATABASES] (4872)

156 remove duplicates from 155 (3418) [TOTAL UNIQUE RECORDS]

157 156 use medall [MEDLINE UNIQUE RECORDS] (1858)

158 156 use emczd [EMBASE UNIQUE RECORDS] (1533)

159 156 use cctr [CENTRAL UNIQUE RECORDS] (26)

160 156 use coch [CDSR UNIQUE RECORDS] (1)
